# Ideas of Self and Adulthood in Girls with Eating Disorders

**DOI:** 10.1192/j.eurpsy.2022.1488

**Published:** 2022-09-01

**Authors:** D. Dovbysh, D. Kurganskaya

**Affiliations:** Federal State Autonomous Educational Institution of Higher Education I.M. Sechenov First Moscow State Medical University under the Ministry of Health of the Russian Federation (Sechenov University), Pedagogy And Clinical Psychology, Moscow, Russian Federation

**Keywords:** eating disorder, self-representation, adult image

## Abstract

**Introduction:**

Adolescence, when physical body image changes occur, is highly vulnerable to the development of eating disorders. At this age, there is an acute task of accepting oneself as another - an adult who has changed.

**Objectives:**

To study the features of the image of an adult in young people with eating disorders.

**Methods:**

The study involved 58 girls (from 17 to 22 years old). The main group included 31 people with a high risk of eating disorders, the control group - 27 people with an average and low risk. Respondents filled in: Taylor Manifest Anxiety Scale, Eating behavior rating scale, projective drawing of an adult and child, association test about words «adult» and «child»

**Results:**

1. A high level of personal anxiety was revealed in the main group; 2. The visualized image of an adult in the main group has more distortions and fewer signs of gender identification than in the normal group; 3. Semantic ideas about adulthood in the main group are negatively emotionally colored and include categories related to eating behavior; 4. Semantic ideas about childhood in the main group are more negatively emotionally colored, and ideas about the present are more connected with appearance than in the control group. Semantic ideas about the future in this group are often negatively colored.

**Conclusions:**

Figurative and semantic ideas about childhood, adulthood and about oneself in the present and in the future in girls with eating disorders have qualitative characteristics in comparison with the control group.

**Disclosure:**

No significant relationships.

